# Glucocorticoids, Stress and Delta-9 Tetrahydrocannabinol (THC) during Early Embryonic Development

**DOI:** 10.3390/ijms22147289

**Published:** 2021-07-07

**Authors:** Alexander G. Kuzma-Hunt, Vivien B. Truong, Laura A. Favetta

**Affiliations:** Reproductive Health and Biotechnology Laboratory, Department of Biomedical Sciences, Ontario Veterinary College, University of Guelph, 50 Stone Road East, Guelph, ON N1G 2W1, Canada; akuzmahu@uoguelph.ca (A.G.K.-H.); vtruong@uoguelph.ca (V.B.T.)

**Keywords:** oocytes, embryos, pre-implantation development, THC, glucocorticoids, molecular stress

## Abstract

Elevated molecular stress in women is known to have negative impacts on the reproductive development of oocytes and the embryos prior to implantation. In recent years, the prevalence of cannabis use among women of reproductive age has risen due to its ability to relieve psychological stress and nausea, which are mediated by its psychoactive component, ∆-9-tetrahydrocannabinol (THC). Although cannabis is the most popular recreational drug of the 21st century, much is unknown about its influence on molecular stress in reproductive tissues. The current literature has demonstrated that THC causes dose- and time-dependent alterations in glucocorticoid signaling, which have the potential to compromise morphology, development, and quality of oocytes and embryos. However, there are inconsistencies across studies regarding the mechanisms for THC-dependent changes in stress hormones and how either compounds may drive or arrest development. Factors such as variability between animal models, physiologically relevant doses, and undiscovered downstream gene targets of both glucocorticoids and THC could account for such inconsistencies. This review evaluates the results of studies which have investigated the effects of glucocorticoids on reproductive development and how THC may alter stress signaling in relevant tissues.

## 1. Introduction

### 1.1. Trends in Cannabis Use

Cannabis use is highly prevalent amongst individuals of reproductive age, specifically between the ages of 15 to 35 years [1,2,3]. With recent movements toward its legalization in many westernized Countries, the use of cannabis is expected to increase dramatically along with the concentration of its main psychoactive component, Δ9-Tetrahydrocannabinol (THC) [4]. According to Statistics Canada, 24% and 26.9% of Canadians aged 15–24 and 25–34 years, respectively, self-reported cannabis use in the last quarter of 2019 [3]. Similarly, the United States in 2016 reported that 9.9% of women aged 18–44 years were cannabis users and, in 2017, 22% of 18–25-year-olds reported regular cannabis use [2,5]. Of particular concern is the prevalence of cannabis use amongst pregnant women, which appears to also be increasing based on data from the United States and Canada showing that self-reported usage ranges from 2–20% [6,7]. Female cannabis users, who fall within the reproductive age demographic, also use cannabis to combat stress-related symptoms such anxiety, depression, and nausea [5]. The use of cannabis to manage stress-related symptoms has generally been associated with low perceived risks, which is likely upheld by the apparent therapeutic value of the Food and Drug Administration (FDA)-approved cannabis products such as Marinol© [7,8,9]. It has been reported that out of a population of 4971 pregnant women and 882,402 non-pregnant women, ages 18–44, about 70% of each group deemed cannabis use once or twice a week to be “slightly or non-harmful” [7]. Cannabis use among women of reproductive age has also been associated with polysubstance use, poor mental health, and depression [5]. These confounders make it difficult to elucidate the direct effects of cannabis on early development and fertility.

Although THC may result in a temporary inhibition of psychoactive stress [10], the literature presents discrepancies regarding the effects of THC on molecular stress signaling in reproductive organs and tissues. There is evidence to suggest that both stress signaling via circulating glucocorticoids, along with THC, have significant impact on reproductive development parameters such as oocyte maturation, quality, competence, and blastocyst rates [11,12,13,14,15,16,17,18,19,20,21,22,23]. However, there is a lack of research that explores the potential interactions between signaling pathways activated by THC and stress hormones at the level of the oocyte and embryo during early development. In addition, there have been no reports of THC-induced teratogenic effects, irrespective of its ability to cross the placental barrier [24]. Strong correlations between rising cannabis use and stress-related symptoms among women of reproductive age suggests that there is a lack of accessible research available for individuals to make more informed decisions regarding their reproductive health and for physicians to properly guide their patients. Therefore, it is of critical importance to expand and translate research on cannabis use and maternal stress during reproductive years to improve fertility and healthcare practice across the globe.

### 1.2. The Endocannabinoid System, Stress Signaling, and Glucocorticoids in Target Organs

**Figure 1:**
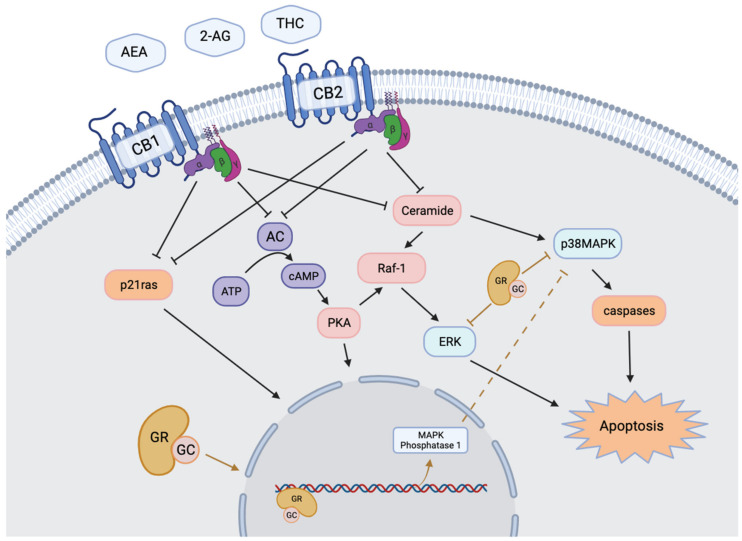
Intracellular regulatory mechanisms stimulated by endocannabinoid and glucocorticoid receptors. Activation of transmembrane cannabinoid receptors CB1 and CB2 stimulates intracellular production of ceramides and inhibition of adenylyl cyclase and p21ras. Activation of membrane bound or cytoplasmic GR results in the cleavage of the peptide into a transcription factor. The two systems converge on transcriptional regulation and modulation of MAPK signaling pathways. Modified from Misner, 2020 [55].

The main components of *Cannabis sativa* and *indica* are cannabidiol (CBD) and THC, that only exerts psychoactive effects upon absorption [11]. Exogenous THC mimics the activity of the two main endogenous cannabinoids (ECBs), 2-arachidonoylglycerol (2-AG) and *N*-arachidonylethanolamine (AEA) by binding as a partial agonist to cannabinoid type-1 (CB1) and type-2 (CB2) receptors [11,25,26,27]. ECBs and their receptors make up the bodies pro-homeostatic endocannabinoid system (ECS), which has been shown to impact almost all reproductive stages in humans and other mammals (Table 1) [13,14,19,23,28,29,30]. Components of the ECS have been discovered in many reproductive fluids, cells, and tissues such as follicular fluid, serum, granulosa cells (GCs), cumulus cells (CCs), oocyte, embryo, ovary, uterus and placenta; however, the distribution of components varies in a species-dependent manner [11,13,14,19,29,30]. The physiological activity of AEA and 2-AG within reproductive tissues are modulated by many enzymes, namely Fatty acid amide hydrolase (FAAH), and Monoacyl lipase (MAGL), which are responsible for breaking down AEA and 2-AG, respectively [11,31]. Intracellular signaling events that follow the activation of CB1 and CB2 receptors generally begin with the inhibition of adenyl cyclase, and end in pathways that control the growth, proliferation, and differentiation of various cell types (Figure 1) [32].

The functions of the ECS are particularly relevant at the level of the female gonads during oocyte maturation and early embryonic development prior to, and following, implantation [13,14,23,28,29,30,31] Specifically, CB1 and CB2 receptors in human GCs of primordial, primary, secondary, and tertiary follicles interact with locally produced AEA in the follicular fluid to ensure oocytes reach adequate sizes during maturation [19] In oocytes, CB1 receptors are critical regulators of intracellular cAMP, which needs to be reduced for normal resumption of meiosis during oocyte maturation [28]. G-protein coupled receptor 55 (GPR55) is another ECS component and target of endogenous cannabinoids, which has been shown to facilitate the formation of normal spindles during the meiotic arrest of oocytes [28]. The successful production and subsequent development of an embryo from competent oocytes is dependent on many factors that are influenced by the ECS. Namely, CB1 receptors have been localized on the outer mitochondrial membrane, suggesting that the ECS regulates the utilization of cellular energy and apoptosis [24,33,34,35]. The direct roles of the ECS during embryonic development is yet to be fully elucidated; however, multiple studies have suggested that the ECS influences embryo development directly and indirectly by modulating gene expression and mitochondrial processes related to apoptosis and cellular energy balance [23,24,33,34,35,36].

**Figure 2:**
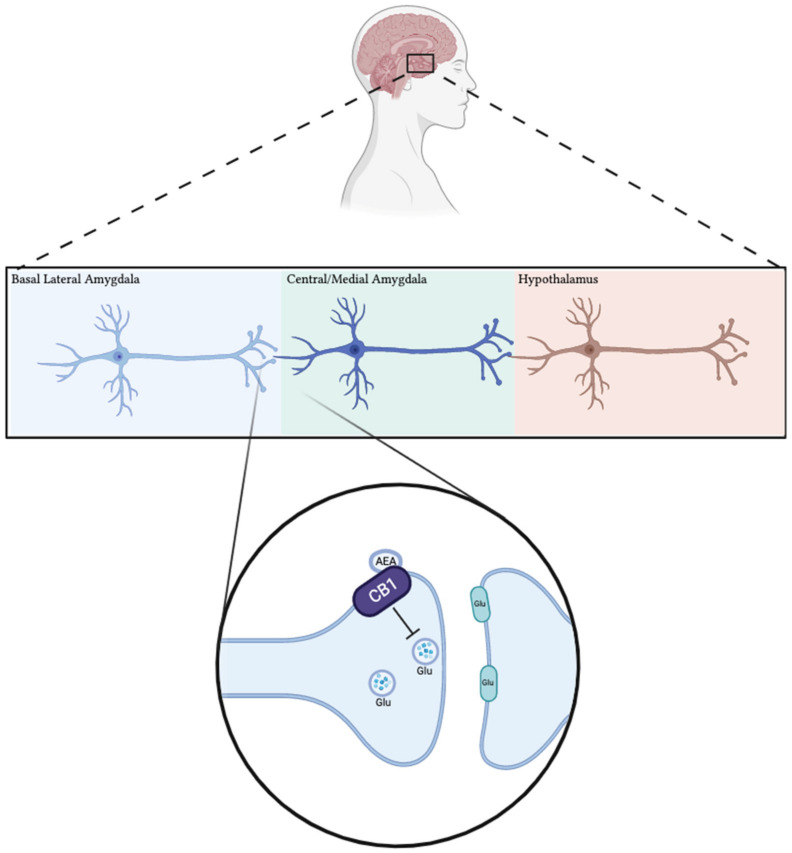
Cannabinoid receptors in the basolateral amygdala (BLA) modulate physiological stress response from the HPA by acting as a brake on glutamate release. Under basal conditions in BLA neurons, inhibitory cannabinoid receptors are activated by high levels of synaptic endocannabinoids, causing inhibition of glutamate release to neurons in the paraventricular nucleus of the hypothalamus. Stress conditions cause the hydrolysis of endocannabinoids (via FAAH), which decreases the activation of cannabinoid receptors and relieves inhibition of glutamate release to the hypothalamus.

Along with localized activity in reproductive tissues, the ECS also has an important role in modulating responses to psychological and physiological stress by interacting with the hypothalamic-pituitary-adrenal (HPA) axis [26,27,37,38,39]. Neuroendocrine responses to stress are determined by activity across the HPA axis, which contains both CB receptors [27,37,38,40,41,42,43,44]. Under normal physiological concentrations of 2-AG and AEA, CB1 and CB2 receptors primarily function to maintain homeostasis of the HPA axis (Figure 2) [26,27,37,40]. This is important given that prolonged HPA activity, or chronic stress, can negatively impact the function of many tissues. The effects of CB1 are particularly relevant in terms of HPA activity as it has been identified in all organs that establish the axis (brain, pituitary and adrenal glands) [26,27]. Although ECBs seem to reduce HPA activity following a stress stimulus, THC may interfere with this correlation given that it is an exogenous cannabinoid, and a partial agonist at CB1 receptors [27,45,46]. Consequently, THC has been shown to induce dose-dependent changes in the ECS within brain regions responsible for controlling HPA activity, including hypothalamus and amygdala [38,43,47].

Cortisol is the primary stress hormone of the body; it is a glucocorticoid released by the upper adrenal cortex and can significantly impact the functional capacity of tissues needed for reproductive development. Cortisol concentrations are modulated by the HPA axis, which is activated when an organism is presented with stress-inducing stimuli, such as a threat to personal well-being, nutritional deficiency, environmental changes, and high social demands or expectations [22,38,48]. The impact of glucocorticoids on their target organs is primarily modulated by the local number of glucocorticoid receptors (GRs) and the concentration of active hormone available to bind to the GRs (Table 1) [22,48,49]. NR3C1 is one of the main GR subtypes that has been shown to be expressed in the ovaries of many different mammalian species [17,20,21,22,50,51,52] and is part of the nuclear receptor subfamily ligand-dependent transcription factors [50]. NR3C1 forms a receptor complex upon binding to cortisol, which translocates from the cytoplasm to the nucleus and binds to glucocorticoid response elements (GREs) on DNA to alter transcription and downstream gene expression (Figure 1) [17,50,52,53]. Two main enzymes, 11b-hydroxysteroid dehydrogenase type 1 (HSD11B1) and type 2 (HSD11B2), are responsible for controlling the concentration of active glucocorticoids within target organs [21,22]. HSD11B1 is responsible for reducing inactive cortisone into active cortisol, while HSD11B2 is a dehydrogenase that oxidizes active cortisol to inactive cortisone [21,22]. Across various mammalian species, HSD11s and GRs have been shown to be expressed and regulate the hormonal microenvironment in many of the same cells and tissues as the ECS, including GCs, CCs, oocytes, embryos, follicular fluid, ovary, and placenta [17,21,22,49,50,52,54]. The hormonal microenvironment is tightly regulated throughout oocyte maturation, given that oocyte competence, quality, and capacity to produce an embryo can be altered by disruption to such processes [20,21,22].

Given the capacity of both the ECS and glucocorticoids to alter reproductive processes [12,15,23,24,31,34,49,50,54,56,57], it is important to investigate potential interactions between these molecules and their signaling pathways within reproductive tissues. Both glucocorticoids and THC have been shown to influence multiple signaling pathways involved in oocyte and embryo development including extracellular signal-regulated kinase (ERK1/2), cyclic AMP/protein kinase A (cAMP/PKA), Protein kinase B (PKB)/AKT, and mitogen-activated protein kinases (MAPK) p38 [12,13,14,30,58] (Figure 1). THC has also been shown to directly alter the expression of GRs within brain tissue and trophoblasts of humans and mice depending on concentration and duration of exposure [44,49,59]. However, the relationship between molecular stress and THC signaling is understudied at the level of the female gonads. Therefore, this review explores the current literature to provide a general insight into the effects of heightened glucocorticoid levels on oocyte and early embryo development and whether cannabinoid signaling, via THC, alters these effects.

### 1.3. Animal Models

Given current ethical standards, elucidating the potential mechanisms for THC-induced changes to stress-signaling during human oocyte and embryo development is difficult. Therefore, it is important to evaluate the validity of previously used animal models to study relationships between molecular stress levels and THC in humans. Mammalian animal models previously utilized to study the effects of THC or molecular stress include mice, pigs, goats, lambs, primates, and cow [12,14,15,17,18,20,21,22,49,50,52,60,61,62]. Evaluation of such animal models has revealed that the bovine oocyte, granulosa cells (GC), and CCs are the most translationally relevant to human reproduction [13,63,64,65]. This is because bovine exhibits the most analogous developmental dynamics to humans in the temporal sequence of their in vitro oocyte maturation (IVM) and single ovulatory nature [65,66]. Additionally, the morphology of bovine and human female gametes is similar [65,66], as are the transcriptomes of human and bovine oocytes compared to those of other vertebrates [67,68]. This is valuable when using a comparative approach to study subcellular signaling events and gene expression because greater confidence can be achieved when extrapolating results from animals to humans. This review provides an inclusive overview of the potential outcomes asserted by hormonal stress signaling within reproductive tissues and how THC may alter such outcomes; consequently, it will thus present findings from multiple animal models. However, emphasis will be placed on results acquired using the bovine model given their significance in species-comparative medical research.

**Figure 3:**
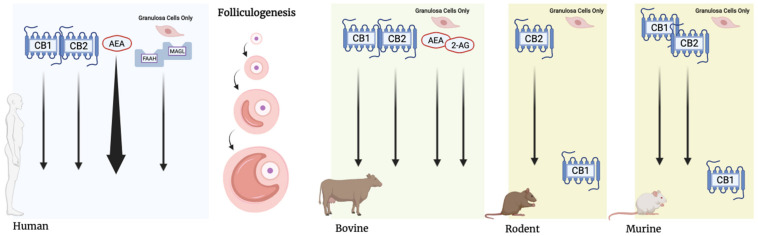
Localization of the endocannabinoid system within ovarian follicles of several mammalian species. CB1 and CB2 are detected in human, bovine, and murine follicles throughout development. Detection of CB1 receptors in rodent specimens have been limited to the granulosa cells of Graafian follicles. CB1 receptors have been detected in the granulosa cells of Graafian follicles in mice. AEA levels in human follicles increase throughout folliculogenesis, peaking in Graafian follicles. Both AEA and 2-AG have been detected in granulosa cells of all follicles. MAGL and FAAH are detected in granulosa cells throughout folliculogenesis in humans.

When conducting comparative research, it is important to consider major species-differences between the variables under investigation. The ECS has been implicated to have an impact on almost all stages of female reproductive development, given that it is widely expressed within all components of this system [11]. Species-specific expression and distribution of ECS constituents present a challenge when generating a widely applicable model of the system. However, based on current literature, the presence of the ECB within mammalian reproductive development can be holistically viewed as presented in Figure 3. Within humans, the use of immunolocalization has facilitated the identification of CB1 and CB2 receptors in oocytes housed by primordial, primary, and secondary follicles, but only CB2 receptors have been detected following antrum formation [19]. In addition, CB1 and CB2 receptors have been detected in GCs during all stages of follicle development [19]. In rat oocytes, only CB2 receptors have been identified in all stages of development, while their associated GCs lacked both CB receptors [69]. In contrast, studies in mice oocytes and embryos have revealed that CB1 is prominent in the late two-cell stage, while CB2 remains present from the one-cell stage up to and throughout blastocyst development [14,70]. Additionally, both CB receptors have been localized within mouse oocytes and mRNA for both brain- and spleen-type CB receptors has been shown to be present within mouse preimplantation embryos [70]. For both mice and rat follicular cells, only CB1 has been found within GCs of tertiary follicles [14]. In bovine oocytes, the expression of both CB receptors has been demonstrated during maturation, with the exception of CB1 during the metaphase 2 stage (M2); however, using immunolocalization, CB1 receptors were detected at the periphery of oocytes during M2, indicating that they still play a role during this stage [13]. In addition, CB1 receptor agonists have been shown to induced ECS-mediated signaling in bovine GCs, confirming the presence of CB1 within these cells during oocyte maturation [13].

**Figure 4:**
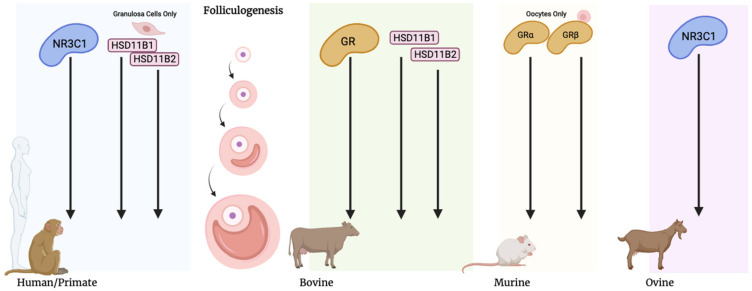
Localization of stress signaling components within ovarian follicles of several mammalian species. Nuclear glucocorticoid receptor NR3C1 has been detected throughout folliculogenesis in human, primate, and ovine models. GRs are also present throughout folliculogenesis in bovine and murine models, though only reported in oocytes in the latter. Enzymes HSD11B1 and HSD11B2 have been detected in human granulosa cells and bovine follicles throughout folliculogenesis.

In terms of stress signaling, genomic analyses of human oocytes collected from primordial and primary follicles showed that glucocorticoid signaling is active during oocyte maturation and early embryo development [71]. In addition, human CCs have also been shown to express HSD11B1, HSD11B2 and GR subtype NR3C1 (Figure 4) [16]. In cows, HSD11B2 and HSD11B1, along with GRs, have been shown to be variably expressed in oocytes and follicular CCs and GCs during all stages of development [21,22,54]. In contrast to cows, the use of immunolocalization in goats has shown the GR variant during development [17]. In primates, immunolocalization has been used to show that NR3C1 is present within oocytes and surrounding follicular cells in vivo and in vitro [50]. Specifically, it was demonstrated that the localization of NR3C1 in primate oocytes matured in vivo, changed from the cytoplasm to the nucleus, while also present on the metaphase plate of metaphase-2 (M2) oocytes [50]. However, primate oocytes that underwent IVM only retained NR3C1 expression and localization in the cytoplasm [50]. In the same study, enzymes HSD11B1 and HSD11B2 were localized in oocytes and GCs and CCs of primates [56]. Studies in rodents have found that cortisol is able to influence the function of GCs, CCs, and oocytes during maturation, suggesting the presence of GRs within these cell types [18,60]. However, it should be noted that the main active glucocorticoid in rodents is corticosterone, which plays an analogous role to cortisol in other mammals [52]. Taken together, components required for both ECS and stress signaling have been shown to be conserved within the reproductive tissues of multiple mammalian species (Figure 3 and Figure 4). Despite many similarities, there are still knowledge gaps regarding the exact mechanisms by which certain mammalian species utilize glucocorticoids and the ECS to regulate oocyte maturation and embryonic development. Species-differences in gene expression, receptor localization and enzyme concentrations within local tissues may appear in a stage-dependent manner and, therefore, should be considered when carrying out species comparative research.

## 2. Impacts of Molecular Stress on Oogenesis and Early Embryonic Development

### 2.1. The Effects of Glucocorticoid Exposure on Oocyte Maturation and Early Embryonic Development

Oocyte maturation is a complex process that involves termination of the meiotic arrest in prophase 1, allowing for further development into metaphase 2 [72]. Ensuring proper progression through this stage is necessary for normal ovulation, oocyte competence, fertilization and subsequent ability to produce an embryo [72]. Oocyte maturity can be determined by tracking molecular and morphological characteristics that define each stage of development. The general stages of oocyte maturation, in order, are germinal vesicle (GV), germinal vesicle breakdown (GVBD), metaphase 1 (M1) and metaphase 2 (M2) [73,74]. Oocyte maturation is regulated by a diverse range of signaling pathways, that are differentially activated across various species and usually culminate in the expression of maturation-promoting factor (MPF), which is necessary for oocyte maturation [30]. ERKs belong to the mitogen-activated protein kinase (MAPK) family and have been shown to become activated in driving oocyte maturation, in concert with MPF, across multiple species [13,14,15,30,61,75]. ERK activation is dependent on phosphorylation of threonine and tyrosine residues during oocyte maturation around GVBD [15]. Expression of ERKs 1 and 2 proteins have also been reported in oocytes collected from primordial and primary follicles in humans [71]. Recent literature has implicated the role of another member of the MAPK family, p38, during in vitro maturation of porcine oocytes [61]. Expression of activated p38 was lowest during the GV stage, rose toward the GVBD stage and eventually hit a plateau throughout the rest of IVM [61]. This study further substantiates the role of active MAPK proteins as a necessary part of oocyte maturation across species. In addition to MAPK proteins, protein-kinase B (Akt/PI3K) signaling has also been implicated as a key regulator of oocyte meiotic resumption given that PI3K activity is required for polar body extrusion and spindle organization [14,30]. Many of the molecular regulatory mechanisms for oocyte maturation are yet to be fully understood; however, drawing connections between current research is the first step in identifying novel and potentially pivotal pathways for developmental success.

Following maturation, an oocyte can form a viable embryo upon fertilization by sperm [74]. The embryo then undergoes cell cleavage, eventually forming a morula consisting of 16 to 32 cells, which subsequently undergoes cavitation and develops into a blastocyst composed of approximately 100–200 cells [76,77]. Cells within the blastocyst are divided into the inner cell mass (ICM) and trophectoderm; this stage ends the period of pre-implantation development, and the following steps of pregnancy involve hatching and implantation into the uterus [76]. Blastocyst quality can be assessed in various ways, including genetic markers and physical characteristics such as cell number, the cohesiveness of the ICM and trophectoderm, and the expansion and volume of the blastocoel [73]. However, the environment in which the oocyte matures and reaches competency, either in vivo or in vitro, also plays a large role in determining the success and quality of the embryo. It is of particular importance to investigate how changes to the follicular environment may impact functional processes involved in oocyte and embryo development such as metabolism, hormone synthesis, and cellular interactions [50]. Therefore, studying embryonic quality in relation to changes in the oocyte maturation environment may serve to provide useful insight in determining the effects of various agents on reproductive success.

Gonzalez et al. (2010) explored the effects of in vitro exposure to glucocorticoids on oocyte maturation and activation of signal-regulated kinases, ERK 1 and 2, in lamb oocytes. [15]. Oocytes were subjected to 24-h maturation in cortisol-supplemented media and visual assessment of maturation progression showed that cortisol only inhibited oocyte maturation at a high dose of 250 µM. However, there were no observed effects to parthenogenic activation in these cortisol-treated oocytes and no effect on subsequent embryo cleavage following IVF [15]. The same group also demonstrated that cortisol reduced the phosphorylation levels of ERK-1 and ERK-2 in a concentration-dependent manner when added to a culture medium during IVM of lamb oocytes [15]. Results showed significant inhibition of ERKs 1 and 2 (non-phosphorylated ERKs) in media containing 2.5–250 µM of cortisol. The role of ERKs in oocyte maturation was further confirmed by using the same molecular methods to show that, irrespective of their presence, no activation of these proteins occurred in immature oocytes prior to maturation. This finding is consistent with other studies that demonstrated a similar role of ERK1/2 activation, along with AKT and their downstream phosphorylation activity during IVM of mouse and bovine oocytes [13,14,30,75,78].

Similar findings to Gonzalez et al. (2010), were documented by da Silva et al. (2018), who investigated the effects of cortisol on bovine oocytes during IVM, and further embryonic development after IVF [12]. However, unlike Gonzalez et al. (2010), da Silva et al. (2018) identified that exposure of immature oocytes to several concentrations of cortisol in the maturation medium resulted in a concentration-dependent decrease in maturation and subsequent cleavage rate [12]. They assessed maturation progression after a 22–24 h period in which oocytes (*n* = 412) were incubated in cortisol at concentrations of 0 (control), 50, 150 and 250 µM [12]. Therefore, the differences in the degree of inhibition found between Gonzalez et al. (2010) and da Silva et al. (2018) are likely due to the use of different concentrations of cortisol exposure or species differences in the regulation of oocyte maturation. Matured oocytes employed for IVF (*n* = 1027) displayed a negative dose-dependent response to cortisol, where fewer embryos progressed to the blastocyst stage when higher cortisol concentrations were present during maturation. In support of the study by Gonzalez et al. (2010), one of the proposed mechanisms for the notable inhibition of oocyte maturation and subsequent development, was the reduced phosphorylation of signal-regulated kinases, ERK 1 and 2 [12]. Given that the MAPK pathway appears to be a molecular target of cortisol [15], this could ultimately explain findings regarding its deleterious effect on oocyte maturation and embryonic development. However, it should be noted that the MAPK pathway is targeted by many modulators other than cortisol, so further investigation of this mechanism is warranted.

Pontes et al. (2019) confirmed the involvement of glucocorticoids during early follicular development by demonstrating stage dependent changes in localization of GR variant NR3C1 in goat ovarian follicles [17]. Their results indicated strong expression of NR3C1 in oocytes of primordial and antral follicles; however, NR3C1 was not present in the GCs of primordial follicles. This supports that cortisol may affect oocyte development in primordial follicles by directly interacting with the oocyte rather than the surrounding follicular cells (GCs). Staining for NR3C1 in granulosa cells revealed a progressive increase in expression from primordial to primary and secondary follicles [17]. At the antral follicle stage, staining for NR3C1 in the granulosa cells, along with the oocyte cytoplasm and nucleus, was strong. Therefore, it can be speculated that the antral follicle stage of development is the most susceptible to changes in glucocorticoids in goats. Additionally, immunolocalization results demonstrated that, upon binding to cortisol, GR NR3C1 becomes a transcription factor, indicated by its cellular localization at different stages of oocyte development. In terms of folliculogenesis, a significant reduction in the production of preantral follicles was observed when exposed to 10 ng/mL of cortisol, compared to other treatments at 0, 1 and 5 ng/mL [17]. Oocytes and follicular diameters were also found to be significantly reduced at all exposure concentrations of cortisol compared to controls. Contrary to da Silva et al. (2018) and González et al. (2010), the activation rates of oocytes increased when follicles were exposed to 1, 5 and 10 ng/mL cortisol post-culture [17]. However, findings related to oocyte quality and development potential were in support of findings from both earlier studies [12,15]. Pontes et al. (2019) illustrated that cortisol plays an important role in pre-implantation and embryonic development, while also highlighting the significance of GR NR3C1 localization throughout these processes [17]. A more recent study by Ravisankar et al. (2021) investigated the association between the level of active glucocorticoids within the follicular fluid and oocyte competency in Rhesus Macaques [50]. They found that the ratio of cortisol to cortisone was significantly higher in the follicular fluid of oocytes that had successfully produced blastocysts following IVF, compared to oocytes that failed to begin cleavage or arrested prior to blastulation. They further supported the role of cortisol in achieving oocyte competence by showing that HSD11B1 was significantly upregulated, while HSD11B2 was downregulated in oocytes, GCs, CCs and theca cells, following ovulatory stimulation in vivo. Additionally, staining for the NR3C1 receptor showed that it translocated from the cytoplasm to the nucleus in oocytes that were matured in vivo, but only remained in the cytoplasm in vitro. Interestingly, they also demonstrated that NRC31 was required for the expansion of CCs by showing that NRC31-knockout COCs failed to expand and release hyaluronic acid, which is required for CC dispersion and cellular reorganization during cumulus-oocyte expansion [50,79]. Taken together, data collected from Ravisankar et al. (2021), evidenced that glucocorticoids may play an important role during COC expansion by binding NR3C1, and that the ratio of cortisol to cortisone in follicular fluid was an accurate indicator of oocyte competence in primates [50]. These findings contradict Pontes et al. (2019) who suggested that elevated cortisol concentrations were associated with a reduction in the diameter and quality of both oocytes and follicles in goats. It is possible that such discrepancies may be a result of species differences in the regulation of cortisol in pre-ovulatory follicles. However, it appears as though NRC31 is involved in glucocorticoid-regulated changes to oogenesis and follicogenesis in both goats and primates. Although there are inconsistencies regarding whether glucocorticoids improve oocyte maturation and their capacity to produce an embryo, it is clear the GR signaling plays an important and modifiable role in reproductive tissues. Additionally, differences in NRC31 expression between primate oocytes that were matured in vitro versus in vivo provide evidence that could explain poor IVM outcomes in humans [50].

Research by da Costa et al. (2016) demonstrated beneficial outcomes associated with glucocorticoid exposure in bovine oocytes during IVM [54]. They showed that COCs matured for 20h in media supplemented with 0.1 μg/mL of cortisol yielded higher blastocyst rates post-IVF, compared to controls [54]. This is in agreement with a prior study by Santana et al. (2014), which showed a similar positive correlation, but between dexamethasone (DEX) exposure and in vitro embryo yield from bovine oocytes [80]. Additionally, da Costa et al. (2016) recorded that embryos from oocytes maturated under the same conditions (0.1 μg/mL cortisol) expressed higher levels of glucose transporter I (GLUT1), fatty acid synthase (FASN) and heat shock protein 70 (HSP70) [54]. GLUT1 and FASN are associated with greater carbohydrate and lipid metabolism, respectively, while HSP70 is responsible for cellular tolerance to stress [54]. Although upregulated in embryos, genomic analysis of CCs and oocytes did not follow this trend [54]. This pattern in gene expression was attributed to a potential long-term delay of transcription until embryo development initiation. Interestingly, a study performed by Simerman et al. (2015) revealed the increased expression of other lipid metabolizing genes, specifically lipoprotein lipase (LPL) and hormone sensitive lipase (HSL) in response to cortisol exposure in human CCs [16]. They postulated that LPL and HSL played essential roles in lipolysis during meiotic resumption in oocytes, with an inverse relationship between lipid content in CCs during meiotic resumption and intrafollicular cortisol due to the upregulation of these lipolytic enzymes. At the same time, intrafollicular cortisol concentration was shown to be positively correlated with oocyte maturation [16]. However, according to da Costa et al. (2016), cortisol upregulates genes directed toward lipid synthesis, but only at the start of embryonic development [54]. Taken together, it appears that the genomic and metabolic effects of cortisol are variable and highly regulated based on stage of development. Assuming that upregulating FASN results in an abundance of fatty acid production, it can be speculated that cortisol concentrations are positively correlated with lipid content at the blastocyst stage, but negatively correlated with lipid content prior to this. It should also be noted that both Costa et al. (2016) and Simerman et al. (2015) indicated a positive correlation between cortisol exposure and oocyte maturation rate [16,54]. However, the use of different animal models complicates interpretation, since species variation regarding the mechanisms of IVM and expression of GRs throughout development may exist.

On the other hand, a more recent study by Banliat et al. (2019), suggested that cortisol altered the phospholipid profiles of bovine oocytes during IVM and embryo development. In contrast to previous studies conducted in the bovine species [12,54], Banliat et al. (2019) found that cortisol had no significant effect on in vitro development and only altered blastocyst cryotolerance rather than rate of development [81]. This provides further support for a relationship between cortisol concentrations and lipid content during pre-implantation development. Lipid droplets are important resources for signaling and meeting the energy requirements of the embryo during the preimplantation stages of development across many mammalian species [82]. A study by Arena et al. (2021) provides evidence to suggest that lipid storage in the oocyte and embryo is especially important for successful survival during embryonic diapause (ED). ED is a temporary arrest of the embryo that occurs prior to receiving adequate maternal signaling needed for uterine implantation [82]. Without adequate energy stored during ED, it was shown that mouse blastocysts fail to survive [82]. Based on this finding, it can be speculated that cortisol may be able to improve blastocyst survival during ED by upregulating fatty acid producing enzymes such as FASN [54]. However, the length of ED differs in a species-dependent manner; therefore, the lipid balance or energy required to sustain embryos during ED will also change in this respect [82]. When considering the utility of various supplements intended to improve energy availability and metabolism during in vitro embryo development, it is also important to keep in mind the “quiet embryo hypothesis” [83]. The “quiet embryo hypothesis” states that pre-implantation embryo development in vitro is best supported by reducing concentrations of nutrient supplements in culture media to promote the use of endogenous resources [83]. This hypothesis has been supported by more recent work by Ravisankar et al. (2021), who used an extensive metabolomics analysis to show that all significant metabolic pathways were downregulated in primate embryos that successfully formed blastocysts compared to those that arrested prior to the blastocyst stage [50]. Taken together, the metabolic requirements of embryos in vitro are very sensitive to changes in nutrient concentrations and factors that may activate oxidative metabolic pathways [83]. Therefore, caution should be taken when considering the use of media supplements such as glucocorticoids to improve embryonic metabolism. Future research should consider investigating the relevant metabolic pathways specified in Ravisankar et al. (2021), given that they report a number of unknown biomolecules that appear relevant to embryogenesis.

### 2.2. Effects of Glucocorticoids on Apoptotic Pathways and Cell Viability during Occyte Maturation and Early Embryo Development

Apoptosis is a form of programmed cell death in which the main effector enzyme, caspase-3, becomes active and degrades cellular and nuclear substrates required for cell structure and function [84]. Activation of apoptosis is dependent on a fine balance between pro-apoptotic factors and anti-apoptotic factors such as the expression of Bax and Bcl-2, respectively [84]. The success of follicogenesis and embryogenesis are dependent on normal expression levels of such factors that regulate cell proliferation and cell death [84]. Consequently, if pro-apoptotic factors are increased to certain thresholds as a result of changes to cellular microenvironments, follicles may undergo atresia or embryos may undergo developmental arrest [18,52,60,85]. Mitigating apoptosis is also relevant in oocytes during maturation or cryopreservation, given that it will impact their competence to successfully produce embryos [18,52,60,85]. Both endogenously and exogenously produced glucocorticoids have been shown to alter apoptotic events and cell viability in mice, rats, and bovine [18,52,60,80,86,87]. Glucocorticoid-induced apoptosis has been shown to act through various mechanisms, including the upregulation of Fas receptor Fas ligand (Fas-Fas L) [60] and tumor necrosis factor (TNF)-α [18,86] in oocytes, GCs, CCs, and embryonic cells of rodents and bovine [18,52,60,85]. However, there are inconsistencies in the research regarding what concentrations of glucocorticoids compromise cell viability and how synthetic glucocorticoid administration could impact fertility.

Yuan et al. (2016) provided evidence that cortisol’s negative effects on oocyte development are a result of triggering apoptosis in ovarian cells via the Fas-Fas L system [60]. A mouse model was used to show that cortisol injections (50 mg/kg of mouse body weight) resulted in decreased oocyte development potential and increased apoptosis in mural granulosa cells (MCGs) and CCs. Ovarian Fas L secretion during IVM and Fas expression in MGCs, CCs and oocytes were also increased due to cortisol [60]. Oocyte competence was assessed after IVM over a 24-h period and oocytes from cortisol-injected mice exhibited significantly less meiosis resumption compared to controls, indicated by less extrusion of the first polar body. Embryonic developmental success was determined by cell counts in blastocysts after culturing embryos derived by activated oocytes past the 3 to 4 cell-stage [60]. Involvement of the Fas-FasL pathway was evaluated by inducing FasL mutations in cortisol-exposed mice. These mutant mice had significantly less apoptosis in MCGs and CCs while also exhibiting greater oocyte competence. Another study by Yuan et al. (2020), explored an alternative mechanism for ovarian cell apoptosis as a result of glucocorticoid exposure. Results demonstrated that cortisol injections (50 mg/kg of mouse body weight) inhibited mouse oocyte development and caused MGC apoptosis by activating the TNF-α apoptotic pathway in vivo and in vitro [18]. Oocytes from female mice injected with cortisol in vivo were confirmed to have significantly higher expression of TNF-α and its associated receptors [18]. In vitro experiments in which MGCs were cultured in the presence of corticosterone yielded similar degrees of apoptosis and TNF-α expression [18]. These studies [18,60] suggest that impaired oocyte development is due to the ability of cortisol to activate various apoptotic pathways. However, they did not consider the variable expression of GR receptor subtypes, which depend on the stage of oocyte maturation or embryo development [52].

Čikoš et al. (2019) found similar results to Yuan et al. (2016 and 2020), but suggested that the effects of glucocorticoids on mouse oocyte and embryo development are modulated by the variable expression of GRs at these stages [18,52,60]. Using qPCR, they demonstrated that GRα and GRγ transcripts were highly expressed in oocytes, but not in pre-implantation embryos [52]. Of particular interest, they showed that expression of GRβ, an ortholog of human GR-P, was downregulated in the blastocyst. These findings indicate that the underlying mechanisms of how glucocorticoids influence pre-implantation development may be dictated by the variable expression of certain GR subtypes in a stage-specific manner. This has relevance to the Pontes et al. (2019) study, where a stage-specific pattern of NR3C1 (GRα) expression was detected in goat ovarian follicles. To study the variable outcomes associated with the activation of different GR subtypes, Čikoš et al. (2019) also evaluated the effects of synthetic and endogenously produced glucocorticoids, DEX and corticosterone, respectively, on embryo development. Corticosterone (50–150 μM) did not influence apoptosis, but inhibited embryo development. The effects of dexamethasone (1.5 to 150 μM) resulted in apoptosis in early embryo cells, but with less development inhibition than corticosterone [52]. Taken together, the evidence suggests that glucocorticoids appear to modulate apoptosis and, consequently, development potential in mouse oocytes and embryos prior to implantation. However, to fully elucidate the outcomes of glucocorticoid exposure, attention must be paid to GR expression during the specific stages of development under investigation. Additionally, animals with greater similarities to human reproduction such as bovine species, are required to establish the relevance of these findings in humans.

As mentioned previously, DEX is a synthetically derived glucocorticoid that is clinically administered for various purposes such as immunosuppression [88]. DEX also has utility as a research tool to study the various effects of glucocorticoids in animal models. Using the bovine model, Silva et al. (2017) investigated the effects of TNF-α and DEX on preantral follicle development, survival, ultrastructure and gene expression [86]. Using immunohistochemical techniques, they first identified the presence of elements of the TNF-α pathway in bovine preantral and antral follicles. They demonstrated that ovarian tissues cultured over six days in the presence of TNF-α (10–200 ng/mL) had a decreased proportion of healthy preantral follicles and an increased incidence of cell death. In addition, it was reported that ovarian tissues exposed to DEX (10–200 ng/mL) during a six-day culture period, maintained production of healthy follicles as determined by ultrastructure integrity. Neither in vitro treatment (DEX or TNF-α) influenced primordial follicle activation or the expression of apoptotic genes such as BCL-2, Bax, and p53 [86]. Data regarding the effects of DEX on apoptosis were consistent with some preliminary research, in which DEX (0.1 mg/mL) had positive outcomes associated with developmental kinetics and cell numbers on days 4 and 7 of bovine embryo culture [80]. In contrast to studies performed in other species [17,18,52,60], Silva et al. (2017) demonstrated a potentially beneficial role of synthetic glucocorticoids in maintaining healthy follicular development and cellular structure. These results also demonstrate the significance of the TNF-α system in bovine follicles, suggesting that apoptotic pathways present in cows and mice are similar [86].

Barroso et al. (2020) continued to evaluate the effects of DEX on bovine folliculogenesis by assessing its influence on growth, viability, antrum formation, morphology and ultrastructure of secondary follicles cultured in vitro. DEX (1–1000 ng/mL) exposure over an 18-day culture period did not alter the growth, antrum formation or survival of bovine secondary follicles in vitro [87]. In agreement with the findings of Silva et al. (2017) for primordial follicles enclosed in ovaries, the ultrastructure of follicular granulosa cells of secondary follicles cultured in the presence of DEX (1, 10, 100 and 1000 ng/mL) were well maintained [87]. However, oocytes contained within secondary follicles exposed to DEX concentrations of 10, 100 and 1000 ng/mL had ultrastructural abnormalities such as reduced microvilli and connections between oocyte and GCs, likely as a result of apoptosis. On the other hand, adversities were not present at a DEX concentration of 1 ng/mL, where the integrity of oocytes, follicular cells and their connections were well maintained. Barroso et al. (2020) delved further than Silva et al. (2017) by evaluating the effects of glucocorticoids on the oocytes from secondary follicles, while also demonstrating that lower concentrations are non-harmful and potentially beneficial to preserving quality [86,87].

**Figure 5:**
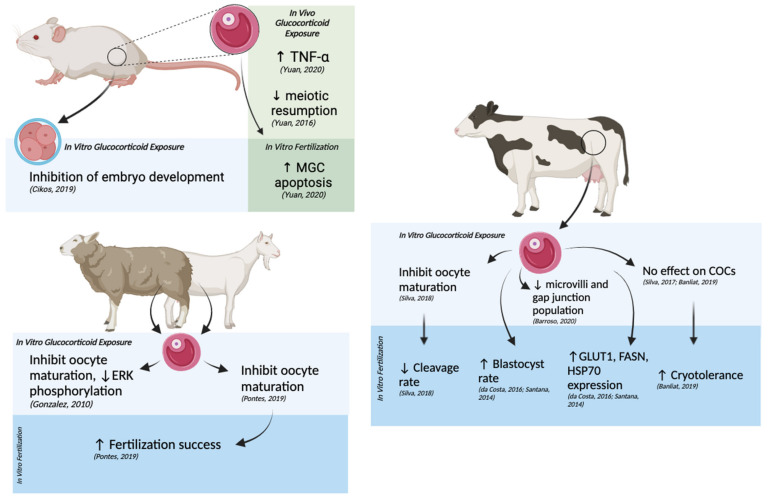
Outcomes of in vivo and in vitro glucocorticoid exposure in oocytes and embryos from mice, lamb, goat, and cow models. Glucocorticoid inhibition of oocyte maturation in vitro is observed in all animal models. Glucocorticoid exposure in mouse embryos inhibited development, while maternal exposure to glucocorticoids caused increased apoptosis in granulosa cells of subsequent embryos. In goats and cows, maternal glucocorticoid exposure increased fertilization success in goats and blastocyst rate in cows, though da Silva et al. (2018) also report a decrease in cleavage rate in cow embryos [12].

The research cited above sheds light on the underlying mechanisms of glucocortocoid-induced effects on oocyte health and early embryo development (Figure 5). Studies also indicate that not all the effects of altering glucocorticoid concentrations during development are harmful and that they may have potential utility in research or medical settings. However, caution should be taken when interpreting the influences of molecular stress across different species given that species differ in physiologically relevant concentrations of glucocorticoids, homeostatic setpoints, the expression of GRs, downstream genes, and glucocorticoid-metabolizing enzymes [20].

## 3. The Effects of THC on Molecular Stress Levels

### 3.1. Influence of THC on Physiological Glucocorticoid Levels and Sensitivity

Cortisol is the main glucocorticoid hormone synthesized and released by the adrenal cortex in response to stress [27,48]. Cortisol concentration in the blood and saliva can therefore be used as an indicator of HPA activity [89,90]. However, due to the diurnal rhythm of cortisol secretion [91], special attention should be paid to the time-of-day samples are collected during in vivo human studies. Preliminary research in humans has suggested that THC raises physiological cortisol levels in a dose-dependent manner [41]. However, according to studies performed in humans and mice, this relationship is also dependent on the duration of exposure [40,41,49,92,93,94]. Specifically, acute exposure to high doses of THC increases circulating glucocorticoids whereas chronic exposure causes a blunted response to stress or atypical HPA activity [40,41,49,92,93,94]. It is unclear whether the latter is a result of over-exposure to the former or if there are alternate contributors. In terms of reproduction, it is important to measure the systemic effects of THC-induced changes to glucocorticoids in the blood serum given that the follicular fluid of late secondary and antral follicles adopts a very similar composition [95]. Therefore, changes in serum glucocorticoids and follicular fluid will likely alter molecular stress signaling in developing oocytes and require more research attention.

Moretti et al. (2014), demonstrated a time-dependent change in circulating corticosterone in mice exposed to 10 mg/kg of THC over a 2-day period versus a 10-day period. Mice exposed to THC over a two-day period exhibited a notable increase in circulating corticosterone, yet mice exposed over a 10-day period showed a significant reduction in corticosterone compared to controls [92]. A similar study conducted in mice by Mayer et al. (2014), identified a similar reduction in HPA axis activity following administration of 5 and 10 mg/kg of THC [93]. The mice experienced stress-inducing conditions prior to THC administration but, in contrast to Moretti et al. (2014), did not experience an increase in corticosterone following acute exposure to THC. Differences in measured hormonal concentrations between the studies of Mayer et al. (2014) and Moretti et al. (2014) may be a result of different timings of corticosterone assessment. Mayer et al. (2014) took measurements one hour after exposure and again at one week [93], whereas Moretti et al. (2014) did so after a 2- and 10-day period [92]. It is possible that 1h is not a sufficient period of time for mice to mount a stress response and raise cortisol concentrations to detectable levels following THC exposure. As another potential explanation for variation in results across the two studies, Mayer et al. (2014) induced stress prior to acute THC exposure, which limits further propagation of its activity as a result of negative feedback. A more recent study performed in rats by Devuono et al. (2020) provides evidence for a dose-dependent increase in serum corticosterone following the administration of THC.

Paradoxical to the use of cannabis as an anti-emetic, Devuono et al. (2020) investigated how the stimulation of the ECS by high doses of THC caused alterations to the HPA axis, which is said to be highly correlated with cannabinoid hyperemesis syndrome (CHS). In groups of eight (*n* = 8), rats were assigned to receive one of three treatments (vehicle, 0.5, or 10 mg/kg THC) for three consecutive days. Rats which received the 10 mg/kg dose of THC had significantly increased serum cortisol levels following the three-day regimen, which is consistent with the findings from Moretti et al. (2014) [38]. They also showed that stress-inhibiting treatments suppressed THC-induced vomiting reflexes (gaping and sustenance aversion) in the rats, while normal anti-emetic drugs did not have the same effect. Therefore, findings by Devuono et al. (2020) support that THC-induced nausea, and CHS, are results of a dysregulated HPA axis following ECS stimulation. According to a survey data from 4735 pregnant women in Hawaii, the 21.2% that experienced extreme nausea during pregnancy were also significantly more likely to use cannabis during pregnancy compared to those who did not experience extreme nausea [8]. Interestingly, the findings from Devuono et al. (2020) provides evidence to suggest that women using cannabis to suppress nausea may actually be exacerbating the issue due to dysregulation of their HPA axis.

King et al. (2011), investigated the effects of chronic cannabis use on various physiological functions, including HPA-activity, in a total of 60 individuals. Their study population included men and women, 30 of whom were cannabis users (6–7 day/week for at least one year) aged 18–45 years, and the other 30 were nondrug users aged 19–44 years [40]. Salivary cortisol was measured as an indicator of HPA activity and results demonstrate that cannabis users had higher cortisol concentrations than non-users. Similar results were found by Kleinloog et al. (2012), who showed that consecutive intrapulmonary administration of THC at 2, 4 and 6 mg, within a 90 min interval, caused an increase in serum cortisol concentration in healthy male subjects aged 18–45 years [96]. Cortisol was measured using electrochemiluminescence immunoassays and displayed a 21% increase in concentration following administration of THC compared to placebo. Consistent with Kleinloog et al. (2012), a study by Klumpers et al. (2012) showed that consecutive inhalation of 2, 4 and 6 mg of THC, within a 90 min interval, resulted in elevated serum cortisol levels in a group of healthy male and females aged 18–45 years, compared to placebo [97]. A recent study conducted by Carol et al. (2017), suggested that THC-related changes in glucocorticoids exist across a diverse demographic of individuals, including those with pre-existing issues related to HPA activity [98]. Salivary cortisol concentrations were collected from 46 patients at risk of psychotic episodes who currently or previously used cannabis in the last month. Results indicated a higher concentration of salivary cortisol in subjects who were existing cannabis users, confirmed by urine screening for THC (cut-off 50 ng/mL), compared to non-users and former users. These results suggest that cannabis has the ability to alter HPA function in individuals with pre-existing mental health problems associated with heightened stress. It is possible that these acute THC-related increases in glucocorticoids are due to the negative allosteric effects of THC on CB1 receptors, which play an inhibitory role in the HPA axis [26]. Furthermore, it can be expected that oocytes and embryos would be subjected to THC-induced increase in circulating glucocorticoids, making these systemic changes to stress hormone levels relevant to the reproductive function.

Other studies have found that chronic cannabis use results in a blunting of the HPA axis in response to stress hormones [94,99]. Somaini et al. (2012) measured changes in serum cortisol concentration among chronic cannabis users, former users, and non-users following the exposure to stress-inducing visual stimuli [99]. Results of blood testing indicate that hormonal responses to stress was suppressed among chronic cannabis users relative to former users and non-users, despite baseline cortisol levels being higher in the chronic-use participants [99]. More recently, Cuttler et al. (2017) presented data that supports a similar relationship between THC and HPA activity in cannabis users [94]. Chronic cannabis users (*n* = 40) displayed significantly reduced reactivity to stress conditions compared to non-users (*n* = 42), as measured by salivary cortisol levels following Maastricht Acute Stress Testing [94]. The above data present the potential for both positive and negative outcomes associated with a blunted stress response. It is important to note that both subjective and physiological stress responses were evaluated in individuals from both studies, allowing for the validation of self-reported stress symptoms [94,99]. Therefore, individuals using cannabis to suppress psychological stress symptoms, such as anxiety, may be creating further issues related to dysfunction of the HPA axis.

The impairment of cortisol mobilization following chronic cannabis use raises important questions regarding the potential effects on reproduction. Cuttler et al. (2017), indicated that cannabis-related suppression to the physiological stress response could serve a beneficial role in enhancing resilience to outcomes associated with elevated stress signaling [94]. Consequently, it can be speculated that prolonged administration of cannabis, or THC, may have the ability to mitigate stress-induced anomalies in reproductive processes. This outcome could be of particular importance to individuals suffering from persistent hyperarousal of their HPA axis, which has been shown to cause infertility [100], and impact oocyte maturation and embryonic development [12,15,52]. However, this field requires further exploration as the literature is still inconclusive on the exact mechanisms and targets of THC in the suppression or stimulation of the HPA axis. Currently, there is mounting evidence to support that acute exposure to THC elevates stress levels and, overtime, reduces glucocorticoid release. According to Newsom et al. (2020), this relationship likely applies to both constitutive and stimuli-dependent HPA activity, which are bi-directionally regulated by interactions between CB1 receptors and its respective ligands [26]. Therefore, the adversities of THC administration outweigh the prospective benefits, according to contemporary research. Future research should consider the effects of various doses of THC, length of use, and genetic or alternative predisposing factors of stress reactivity in users and non-users to further elucidate the influences on HPA axis activity.

### 3.2. Effects of THC on Glucocorticoid Receptors and Downstream Gene Expression

Previous research has shown that the expression of certain downstream signaling targets of GRs such as HSP70, FASN and GLUT1 could be altered due to in vitro conditions and are key determinants of embryo quality [54]. Upon binding to glucocorticoids, GR-hormone complexes translocate to the nucleus and either activate or repress key genes associated with reproductive development, including GLUT1 [54]. GLUT1 is the primary glucose transporter during embryogenesis and is upregulated in bovine embryos following cortisol-induced stimulation of GRs during COC IVM [54]. Interestingly, Natale et al. (2020) showed that THC could directly interfere with this process by reducing the number of GRs and steady state levels of mRNAs for GRs and GLUT1 in reproductive tissues. Following in vivo exposure to THC (3 mg/kg) during gestation, GLUT1 expression and positive staining for GRs were reduced in the labyrinth layer of rat placentas compared to controls. Given that the labyrinth is the site of maternal-fetal nutrient exchange, diminishing the ability of this tissue to uptake glucose via GLUT1 was attributed to previous associations between THC consumption during pregnancy and intra-uterine growth restriction [49]. However, THC can elevate circulating glucocorticoids, and prolonged exposure to these hormones also results in the downregulation of GRs [38,101]. Therefore, it can be speculated that chronic maternal exposure to THC may result in elevated glucocorticoids, causing reduced GR expression [49].

To investigate alternative explanations for the effects of THC on GRs and GLUT1, Natale et al. (2020) also cultured human BeWO trophoblast cells in media containing 15 µM of THC, 15 µM 11-COOH-THC, or no THC (control) for 24 h. The use of qPCR revealed a significant reduction in steady-state levels of mRNA for GLUT1 and GRs in the THC group, but no significant change in the 11-COOH-THC metabolite. These results provide evidence that THC, rather than its metabolite (11-COOH-THC), has a direct effect on GR expression in human trophoblast cells, which make up the trophectoderm layer of blastocysts [49,102]. This interaction is of major significance, given that GLUT1 is widely expressed in reproductive tissues and considered a molecular determinant of successful embryo and fetal development in rodents, bovine, and humans [49,54]. The novelty of this study is furthered by the fact that they provide direct support for very preliminary findings from Eldridge and colleagues in the 1990s, which suggested that interactions between THC and GRs in the brain resulted in a time-dependent down-regulation of GRs [44,59]. In reference to the previous section, results from Natale et al. (2020) could, alternatively, be used to support that THC has the potential to be used clinically, or in a research setting, to mitigate stress-induced outcomes in various tissues as a result of its direct inhibitory effects on GR expression. In theoretical application, THC being used to reduce GR expression would require a means of site-specific delivery to limit its actions to a specific location or tissue. However, current literature suggests that the capacity of THC to cause adverse effects during early development outweigh its potential benefits.

HSPs are a family of chaperones that modulate cellular responses to stress by preserving the integrity of proteins and DNA, while also mitigating apoptosis [103]. HSP-mediated processes are especially critical for maintaining cell viability when misfolded proteins begin to accumulate due to the presence of intracellular reactive oxygen species (ROS) [103]. Relating to reproductive function, HSP70 has been shown to be two-fold higher in women compared to men [104] and levels of HSP70 are associated with higher IVF success [105]. Additionally, HSP70 is the most conserved chaperone across all mammalian species, thus, results obtained from animal models is largely applicable to humans [105]. Both THC (20 µM) and Cortisol (0.1 μg/mL) exposure have been shown to increase the expression of HSP70 in human trophoblasts and bovine embryos, respectively [24,54]. These observations provide evidence to suggest that genes encoding for HSP70 may be a common downstream target for both glucocorticoid- and THC-mediated signaling. In contrast to speculations derived from the studies of Natale et al. (2020), Walker et al. (2021) suggested that THC does not buffer subcellular stress signaling through interactions with CB1 and CB2 within the human trophectoderm [24]. They demonstrated that exposure to 20 µM THC significantly increased expression of HSPs 60 and 70, but not antioxidant enzyme mRNA within HTR8/SVneo cells. The increase in HSPs 60 and 70 was thought to be a result of THC-induced mitochondrial dysfunction resulting in the accumulation of ROS, which are normally removed by superoxide dismutase’s 1 and 2 (SOD1 and SOD2) [24]. When ROS build-up within cells and surpass certain thresholds, necrosis, autophagocytosis, or apoptosis occurs, and cell viability is compromised [106]. Unlike Walker et al. (2021), Natale et al. (2020) used a dose of THC (15 µM) that had previously been shown to not compromise cell viability as a result of ROS-induced apoptosis. Interestingly, there has been research to support that short-term and sublethal stress exposures may promote an adaptive and increased tolerance to subsequent stress in bovine oocytes [54,107]. Furthermore, blastocyst and cleavage rates in pig oocytes exposed to sublethal stress condition have been shown to increase [108]. Findings of this nature suggest that the developmental outcomes associated with glucocorticoid and THC exposure are concentration dependent and that either compounds may promote cellular stress tolerance by upregulating HSP70 through various mechanisms in embryos. It appears that THC-induced upregulation of HSP70 could be a result of ROS production or alternative signaling pathways that require further investigation, while cortisol-receptor complexes may directly induce HSP expression following stress induction. Although high ROS exposure is associated with poor developmental outcomes in vitro, physiological levels are required for the initiation of certain process such as reinitiating meiosis in arrested oocytes and activation of specific MAPK proteins [106]. Therefore, the dose-dependent generation of ROS via THC may serve utility in contexts where oocytes fail to exit meiotic arrest.

There is conflicting research regarding the effects of THC alone on IVM and subsequent production of embryos. Studies that have investigated the molecular mechanisms underlying THC-induced changes to oocyte maturation and embryo production provide valuable insight into how THC could influence glucocorticoid signaling during these processes. Many studies have demonstrated that the presence of CB1 receptors on the oocyte and GCs have an integral role during maturation [13,14,30] and that subsequent embryo development is impaired when there is a CB1 deficiency during IVM [14]. It has been shown that exposure to THC during IVM enhanced oocyte maturation in mice (100 nM THC) [14,30] and bovine (0.1 µM THC) [13] by increasing the activation of ERK1/2 and Akt/PI3K, which are genes necessary for proper development. In mouse models, THC exposure during IVM was also associated with an improvement in blastocyst yield [14,30], while in bovine blastocysts it was shown to upregulate quality associated genes including interferon tau (IFNτ) and gap junction alpha-1 protein (GJA1), in a dose-dependent manner [13]. As mentioned previously, cortisol has been shown to act in an opposite manner and reduce the activation of ERK1/2 during the maturation of lamb oocytes [15]. These findings suggest that there may be an overlap between CB1 and GR signaling within oocytes undergoing maturation and that THC may be able to mitigate the adverse effects associated with stress-induced inactivation of ERKs. However, species differences could influence the validity of this speculation and should be taken into account when applying these findings.

The dose-dependent effects of THC on oocyte and early embryonic development could also be due to the fact that EKR1/2 and Akt/PI3K are downstream targets of cellular growth-pathways initiated by epidermal growth factor (EGF) during development [109]. EGF promotes oocyte development by binding epidermal growth factor receptors (EGFR) located on CCs and GCs, which subsequently initiates downstream signaling cascades that activate ERK1/2 and Akt/PI3K [109]. Research has shown that EGRF-induced activation of ERKs is necessary for meiotic resumption during oocyte maturation along with GC proliferation and CC expansion across species [109,110,111]. Therefore, the effects of THC on oocyte maturation and competence may be reflective of altered activation of growth-related pathways. The activation of growth pathways could also explain the dose-dependent effects of THC on oocyte development, given that high doses of THC may result in prolonged overexpression of ERKs, which has been shown to result in apoptosis [112], while lower doses of THC may enhance normal development. However, the studies that investigated the effects of THC on ERK/MAPK signaling in bovine [13] and mouse [14,30] supplemented the oocyte maturation media with 10 ng/mL of EGF, suggesting that THC exerts its downstream effects in the presence of growth factors. Moreover, Lopez et al. (2016) suggested that THC played a similar role to EGF during bovine oocytes IVM, given its ability to promote maturation by increasing the amount of activated ERK1/2 [13]. Taken together, further investigation is required to elucidate the exact mechanisms for how THC may impact ERK/MAPK activation given that they are a downstream target of both growth-factors and stress signaling. Alternative growth routes, such as those initiated by platelet activating factor (PAF), should also be considered given its capacity to modulate cellular proliferation and differentiation through ERK/MAPK gene expression [113]. However, research appears conflicting on whether or not PAF and EGF supplemented media equivocally enhance the development of bovine and mouse embryos in vitro, suggesting that species variability exists in terms of optimal growth pathway activation [114,115]. Therefore, future research should ensure that growth-factor concentrations are species-specific, despite the equivalent doses utilized in the studies previously discussed [13,14,30].

On the other hand, more recent investigation into the effects of THC on oocyte competence and early embryo development provide evidence that THC-exposure during IVM does not enhance development [23]. Misner et al. (2021) showed that increasing THC exposure (0.32 µM and 3.2 µM) concentrations during IVM resulted in a significant dose-dependent reduction in the maturation rate of bovine oocytes and cleavage rate [23]. Additionally, 0.032 µM of THC was shown to reduce the expression of connexin 37 and 43, which are associated with high oocyte competence and embryo quality. In blastocysts that had been produced from oocytes matured in THC (0.32 µM and 3.2 µM), there was a significant increase in apoptotic cells relative to control [23]. These findings are consistent with preliminary research by Paria et al. (1995) who showed that THC (10 µM) arrested mouse embryonic development in vitro [70]. Findings from Misner et al. (2021) further support that the negative effects of THC on oocyte maturation and embryo production overwhelm its potential utility for modulating stress or promoting growth in vitro [23].

## 4. Conclusions: Potential Interactions between Glucocorticoids and THC at the Systemic, Cellular, and Molecular Levels

**Figure 6:**
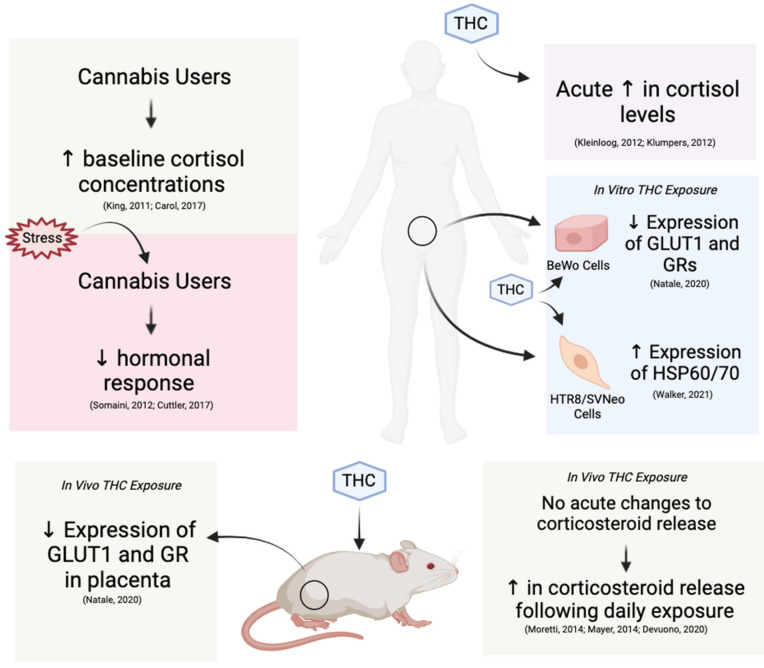
Systemic, cellular, and molecular changes to glucocorticoid and glucocorticoid receptors following THC exposure in humans and mice. Chronic cannabis usage in humans increases baseline cortisol levels and decreases reactivity to stress. Both humans and mice experience an increase in glucocorticoid levels following THC exposure. Decreased expression of GLUT1 and GRs have been observed in both species in response to THC.

This review outlines the possible implications of THC-dependent changes in glucocorticoid hormones and how such changes may alter oocyte maturation and early embryo development. Current research suggests that there are various potential interactions between THC and stress signaling at the systemic, cellular, and molecular levels (Figure 6). Elevated molecular stress mainly exerted harmful effects such as arrest of oocyte maturation and embryo development, apoptosis in CCs and GCs and compromised reproductive morphology [12,15,17,18,52,60,87]. Research regarding the effects of THC on oocyte maturation and subsequent embryo development revealed similar downstream targets to glucocorticoid signaling, such as the MAPK pathway, HSP70 and GLUT1 expression, suggesting that interactions can occur between the two [13,14,15,24,30]. Studies have also indicated that there is potential for both positive and negative effects related to THC-dependent changes in glucocorticoids in clinical and/or research settings.

Elevated exposure to glucocorticoids has been reported to impair oocyte maturation and subsequent embryonic development via reduced phosphorylation of ERKs [12,15]. However, multiple studies have shown that THC can promote the phosphorylation of ERK 1/2 and Akt, which subsequently drive the maturation of oocytes and embryonic development [13,14,30]. Additionally, THC exposure in vivo and in vitro has been shown to reduce sensitivity to localized and systemic glucocorticoid signaling, both directly and indirectly, in a time- and dose-dependent manner [26,44,49,92,93,94,99]. Finally, THC has also been shown to upregulate genes associated with gap junctions in the cumulus-oocyte-complex (COC) [13]. The presence of gap junctions in the COC is essential for the expression of HSD11B1, which is required to maintain the glucocorticoid microenvironment during oocyte maturation [21,22]. Therefore, THC-induced stimulation of the ECS could indirectly contribute to modulating glucocorticoid concentrations by maintaining the structural integrity of the COC during IVM. Taken together, there is evidence to suggest that THC may buffer negative glucocorticoid-related outcomes in reproductive tissues.

On the other hand, acute exposure to THC was shown to elevate circulating glucocorticoids [38,92,96,98], which pose stress-related risks to reproductive development. Elevated exposure to glucocorticoids during oocyte maturation and embryonic development may result in developmental arrest [12,15,18,52,60], compromised morphology [17,87] or apoptosis [18,52,60]. THC-induced downregulation of GRs only presents potential use in cases where there is hyperarousal of the HPA axis such that GRs are being over stimulated. In studies that investigated this interaction, THC was attributed to causing a direct downregulation of GR-associated genes such as GLUT1 [49]. Given that GLUT1 is the main glucose transporter present in the oocyte, embryo and placenta, THC-induced reduction in its expression is likely to interfere with the metabolic requirements associated with early reproductive development [49]. THC has been shown to compromise oocyte maturation, cleavage rates and blastocyst quality [23]. Additionally, THC was suggested to induce the intracellular generation of ROS in the absence of increasing antioxidant enzymes in placental cells [24]. In contradiction to the common justifications for cannabis use amongst individuals of reproductive age, THC appears to exacerbate stress-related conditions related to HPA-dysfunction, which may induce nausea and CBS [38,43]. Consequently, it appears as though the adversities associated with the consumption of cannabis and THC-related changes to glucocorticoid signaling outweigh its therapeutic capacity in both clinical and recreational settings.

Although elevated glucocorticoids seem to have negative repercussions on reproductive development, discrepancies still remain. Specifically, there is evidence to suggest that glucocorticoid exposure is associated with elevated blastocyst rates [54,80], higher expression of proteins associated with stress tolerance, and carbohydrate and fat metabolism [16,54]. Variability in these outcomes across studies could be attributed to inconsistent cortisol dosing in vitro and in vivo. Additionally, species differences between the expression of glucocorticoid metabolizing enzymes have also been identified as a contributing factor to inconsistencies in the literature [20]. To address such inconsistencies, future research should establish and apply standardized doses of both THC and glucocorticoids based on relevant physiological concentrations in humans or the animal model being used for the investigation. Additionally, dosing of THC should parallel blood concentrations occurring with common routes of administration, such as smoking, intravenous delivery or oral ingestion. Given that the effects of THC are also time-sensitive, attention must be paid to the duration of exposure and how chronic versus acute timelines are defined. With respect to stress signaling, further investigation should consider predisposing genetic, or other factors to stress sensitivity in reproductive tissues. Furthermore, there is a lack of data from humans on this topic, which emphasizes the importance of using translationally relevant animal models in research. Lastly, many of the studies cited in this review do not investigate the molecular interactions between THC and glucocorticoid-induced stress within the same experimental context. Therefore, future research should focus on the reproductive effects of THC-related changes in glucocorticoids as a primary outcome within oocytes and surrounding tissue.

To conclude, current research shows various potential outcomes associated with elevated molecular stress on oocyte maturation and embryonic development in mammalian species, including humans. The literature presents implications related to the use of THC and how this could potentially alter stress signaling. Although some studies demonstrated harmful consequences following gamete exposure to glucocorticoids, the results were variable due to dose, species differences and duration of interventions. Therefore, results should be interpreted with caution, given that some may be species-dependent or based on irrelevant physiological concentrations. Nonetheless, the findings presented in this review serve as a foundation for future investigation into this topic. Given the increasing THC concentrations and use of legalized cannabis, it is important to advance our understanding of THC-dependent changes in molecular stress and how this affects the reproductive success in a growing number of cannabis users.

## Figures and Tables

**Table 1:** Primary Components of the Mammalian ECS and stress signaling systems.

System	Molecule	Function
**Endocannabinoid System (ECS)**	**AEA** (*N*-arachidonylethanolamine, anandamide)	Endocannabinoid
	**2-AG** (2-arachidonylglycerol)	Endocannabinoid
	**CB1**	Cannabinoid receptor
	**CB2**	Cannabinoid receptor
	**FAAH** (Fatty acid amide hydrolase)	Metabolize AEA into arachidonic acid and ethanolamine
	**MAGL** (Monoacylglycerol lipase)	Metabolize 2-AG into arachidonic acid and glycerol
**Stress Signaling**	**Cortisol**	Endogenous stress hormone
	**Corticosterone**	Endogenous stress hormone (*murine only*)
	**Glucocorticoid Receptor**	Cortisol receptor
	**HSD11B1** (11β-Hydroxysteroid Dehydrogenase Type 1, cortisone reductase)	Convert biologically inactive cortisone into active cortisol
	**HSD11B2** (11β-Hydroxysteroid Dehydrogenase Type 2)	Convert biologically active cortisol into inactive cortisone

## Data Availability

Not applicable.
